# Application of an Acoustic Doppler Velocimeter to Analyse the Performance of the Hydraulic Agitation System of an Agricultural Sprayer

**DOI:** 10.3390/s18113715

**Published:** 2018-11-01

**Authors:** F. Javier García-Ramos, Jorge Badules, Antonio Boné, Emilio Gil, A. Javier Aguirre, Mariano Vidal

**Affiliations:** 1 Escuela Politécnica Superior, University of Zaragoza, 22071 Huesca, Spain; jbadules@hotmail.com (J.B.); anbone@unizar.es (A.B.); vidalcor@unizar.es (M.V.); 2 Departamento de Ingeniería Agroalimentaria y Biotecnología, Universitat Politecnica de Catalunya, 08860 Castelldefels, Spain; emilio.gil@upc.edu; 3 CPIFP Montearagón, 22071 Huesca, Spain; angeljavieraguirre65@gmail.com

**Keywords:** fluid velocity, nozzle, tank, copper oxychloride

## Abstract

An acoustic Doppler velocimeter (ADV) was used to analyse the impact of an agricultural sprayer’s agitation system settings on fluid velocities inside the tank. A 3000 L capacity sprayer equipped with a 4-nozzle hydraulic agitation system was used. ADV measurements were carried at 32 points inside the tank under the following settings: circuit pressures of 8, 10, or 12 bar; water level in the tank of 1000, 2000, or 3000 L; 2 or 4 active nozzles. An agitation test with a concentration of 0.4% copper oxychloride was employed to analyse the concentration of active matter as a function of tank fill level and number of active nozzles. All parameters significantly affected the fluid velocity, which increased with increasing pressure, but decreased with increasing water level in the tank and an increased number of active nozzles. Concentration tests showed greater active matter concentrations when higher velocities were recorded by the ADV. The ADV was shown to be a useful tool for the rapid assessment of fluid velocities; in the future, it could be used to validate the design of agitation systems, and to estimate their capacity to ensure an adequate level of active matter concentration in the fluid.

## 1. Introduction

Air-assisted sprayers used in fruit orchards are designed to distribute a uniform dose of pesticide over the entire canopy. Many researchers have studied the influence of the main working parameters on treatment performance, including air flow, fluid pressure, nozzle type, volume of vegetation, forward velocity, etc. To date, the analysis of these parameters has assumed that the concentration of active matter in the tank is uniform. To guarantee this uniformity, tanks of agricultural sprayers are normally equipped with hydraulic agitation systems.

These systems consist of one or several nozzles (jet agitation systems) which, working at a specific pressure, introduce a flow rate into the tank, generating turbulent flow. The mixing quality depends on different factors, including the geometry of the tank, the quantity of water in the tank, nozzle locations, nozzle flow rates, system pressure, and the time available for mixing.

Currently, the experimental procedures used for evaluating the mixing quality of the agitation systems in new agricultural sprayer tanks are based on the standard ISO 5682-2 [1,2], which specifies the methods of testing and assessing the performance of agitation systems in hydraulic sprayers. This standard requires, after a fixed mixing time, sampling from several locations to measure the concentration of active matter. For the case of sprayers in use, ISO 16122 [3] requires only a visual assessment.

Alternative methods to evaluate the performance of agitation systems have been considered by different studies [4] and fall under two different approaches: the assessment of liquid turbulence inside the tank using three electronic flow meters and measurement of the concentration of a solid tracer (glass microspheres) mixed in the water. [5] used digital images taken through a transparent tank to analyse the deposition of particles from a suspension of copper oxychloride. In other investigations [6,7,8], several commercial turbidity meters have been tested for potential use in determining mixing efficiency by measuring the concentration of solids in the liquid.

The study of agitation systems should allow for analysing the effect of set variables (pressure, number of nozzles, nozzle flow, nozzle position, liquid level in the tank) on the concentration of active material in the sample. Performing this type of study according to the ISO 5682-2 standard, for different pressure configurations, nozzle types, etc., would be prohibitively expensive and time consuming; instead, equipment manufacturers require rapid measurement methods that can validate the modelling carried out in the design phase. An ideal experimental test method would be one that provides information to allow manufacturers to quantify the same parameters used by engineers in the design phase, which in most cases are the estimated flow velocity at different points of the sprayer tank by using computational fluid dynamics (CFD). For this purpose, different studies have investigated fluid velocities inside spray tanks using CFD, and these values have been validated by experimental measures [9,10,11]. As an additional step in this line of work, some researchers [12] CFD modelled the movement of fluid in the 4000 L tank of an agricultural sprayer and attempted to correlate the velocity of the fluid with the concentration of active matter, obtaining inconclusive results.

Fluid velocities inside the tank provide useful information regarding the operation of the agitation system, and there are different technologies available for velocity measurements, including: particle image velocimeters [13], laser Doppler velocimeters [11], hot-film anemometers [10], acoustic Doppler velocimeters (ADV) [14], electromagnetic current meters [15], and electronic flow meters [4]. In most cases, acoustic Doppler velocimetry has been the preferred method [13,16,17] because it is relatively low cost, can record at a relatively high frequency (up to 100 Hertz (Hz)), can measure three dimensional instantaneous velocity measurements, and is non-intrusive because it has a relatively small sampling volume according to the instrument selected. Furthermore, calibration is invariant [15].

ADVs are commonly used for fluid flow characterization and for the determination of suspended solids and turbulence [14]; they have been used in a habitual way for the measurement of velocities in river beds, lakes, and natural water environments [18]. ADVs operate on the principle of Doppler shift. Two acoustic pulses of different duration are transmitted, separated by a time interval, and the energy backscattered by particulate matter in a sampling volume at a short distance from the probe tip is recorded after each pulse. The velocity is then calculated as a function of the phase lag between the return signals [19]. ADV measurements are contaminated by Doppler-instrument noise [13], or by spikes, which are random outliers that can occur owing to interference of previous pulses reflected from the flow boundaries or due to the presence of bubbles, sediments, etc. in the flow [20]. Therefore, the signal must be carefully analysed and, if necessary, data cleaning techniques applied to ensure signal quality. Nonetheless, research conducted working with ADV velocimeters [21], stated that mean flow measurements may be reliably obtained less than 10 mm from the fluid boundaries.

The use of a three-dimensional velocity measuring device inside the tank of an agricultural sprayer under different working conditions (pressure, number of nozzles, position of nozzles, etc.) would make it possible to know the effect of the regulation parameters on the operation of the system and, in this way, estimate the efficiency of the agitation system on the basis of these conditioning factors. Therefore, the objective of this work was to study the applicability of using an ADV to investigate the operation and efficiency of a hydraulic agitation system in the tank of an agricultural sprayer according to different working parameters.

## 2. Experimental Design 

### 2.1. Tank Characteristics

**Figure 1:**
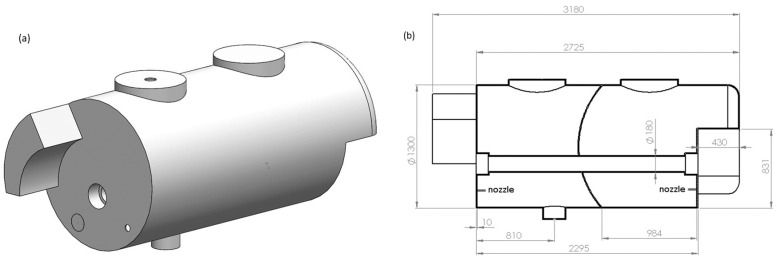
Geometry of the sprayer tank with a 3000 L of capacity. (**a**) Exterior 3-dimensional (D) view; (**b**) Internal longitudinal section, 2D view, with two interconnected parts. Dimensions in millimetres.

This study was carried out using an air-assisted sprayer with a nominal capacity of 3000 L (GarMelet S.L., Huesca, Spain). The geometry of the tank was cylindrical, and the inside was divided into two interconnected parts (Figure 1) separated by a breakwater wall with several orifices to facilitate fluid circulation. The agitation system consisted of four nozzles placed on the bottom of the opposite sides of the cylinder, two in each side. The inside geometry of the tank is shown in Figure 1.

### 2.2. Agitation Nozzles

**Figure 2:**
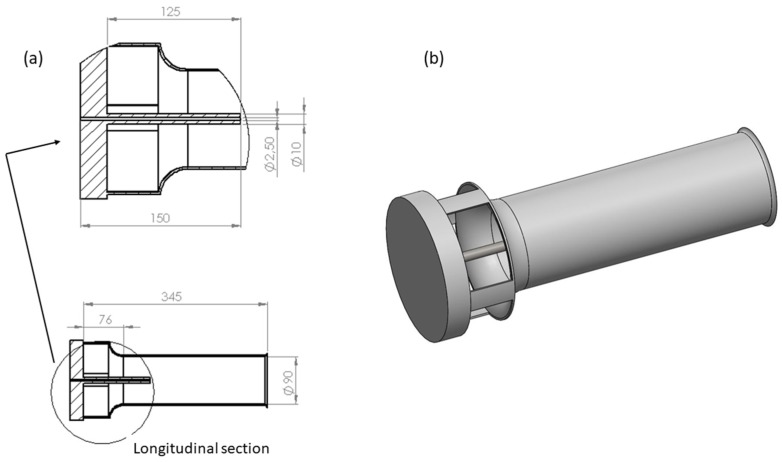
Geometry of the Venturi agitation nozzles. (**a**) Longitudinal section; (**b**) Three-dimensional (3D) view. Dimensions in millimetres.

The nominal flow rate of the agitation nozzles was measured for different working pressures (8, 10, and 12 bars). The sprayer was equipped with four Venturi type hydraulic agitation nozzles (Figure 2). In this sense, the nominal flow rate provided by the nozzle, thanks to the Venturi effect, produced an actual flow rate in the Venturi outlet of approximately 40 times the nominal flow of the hydraulic nozzle located inside the Venturi body.

Table 1 shows the nominal flow rates provided by a single nozzle for the different working pressures considered. The flow rate of the agitation system nozzles was measured in the laboratory. For this goal, the nozzles were disassembled from the machine and the flow rate of a single nozzle was quantified for 30 s at different working pressures (8, 10 and 12 bar). Subsequently, the rest of the nozzles were tested in a similar way to the pressure of 10 bar to check that the nominal flow did not show variations between nozzles. 

### 2.3. Acoustic Doppler Velocimeter

**Figure 3:**
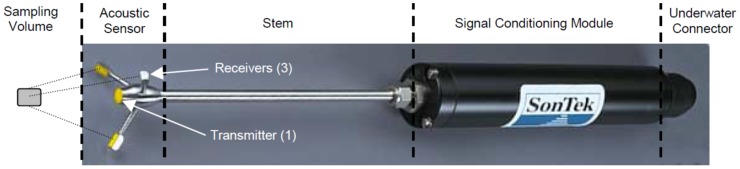
Three-dimensional Sontek Micro acoustic Doppler velocimeter (ADV) 16 megaHertz (MHz).

A 3-dimensional (D) microacoustic Doppler velocimeter (3D MicroADV 16 megaHz (MHz) by Sontek, San Diego, CA, USA) was used to carry out the velocity measurements. The probe head included one acoustic transmitter and three acoustic receivers (Figure 3). The remote sampling volume in which the ADV took velocity measurements was located 5 cm from the tip of the acoustic transmitter. Table 2 shows the technical characteristics of the probe. The MicroADV consists of the acoustic sensor, the stem (or cable) and the signal conditioning module.

### 2.4. Fluid Velocity Measurements

An experimental factorial design was carried out with 3 independent variables for the configuration of the agitation system: water level in the tank (1000, 2000, or 3000 L); number of active nozzles (2 or 4); and working pressure of the agitation circuit (8, 10, or 12 bar).

**Figure 4:**
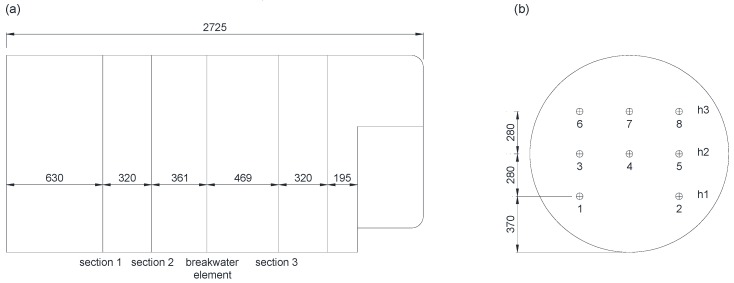
(**a**) Cross sections of where velocity measurements were made inside the tank (dimensions in millimeters); (**b**) Velocity measurement points within each of the four sections.

Velocity measurements were made in four circular sections of the tank, with eight measuring points in each section distributed at three heights (Figure 4), and working with water inside the tank. These measurements were carried out considering the different combinations of the variables (pressure, number of nozzles and tank filling level). In this way, Table 3 reflects the measurements made for the different variable configurations. When the system worked with two nozzles, these were on opposite sides of the tank.

Velocity measurements were taken using the ADV at a frequency of 50 Hz. The agitation system was configured in terms of working pressure, number of nozzles activated, and water level in the tank. A mechanical implement specifically designed to position the ADV at the different measuring points was used. Sections 1 and 2 of the tank were accessed from the front filler neck and Sections 3 and 4 from the rear filler neck (Figure 4). The tank was first filled to the required water level. Subsequently, the ADV was placed at its measuring point. Finally, the parameters of pressure and number of nozzles were established, and the agitation system was left working for 3 min to achieve stabilization of fluid flow. After fluid flow was stabilized, data were collected for 20 s at each measuring point. The procedure was repeated for each measuring point and configuration of the agitation system.

Measures provided by the ADV with a correlation of less than 70% were eliminated [19]. The correlation parameter, which varies from 0 to 100, is an indicator of the relative consistency of the behavior of the scatterers in the sampling volume during the sampling period. ADV’s collect data at a higher sampling rate than the sample reporting period, and the correlation parameter indicates the consistency of the multiple measurements that take place within each sampling period [22]. The signal-to-noise ratio was always greater than 20 dB, such that the signal did not cause increased noise in the velocity data. Under these conditions, noise in data output should be about 1% of the velocity range setting [19].

Finally, data were averaged to obtain a single data per second, thus reducing the number of data to 20 for each measuring point and system configuration. The vector module was taken as the representative value of the velocity, independent of its direction. It is foreseeable that agitation systems that produce velocity flows directed against the bottom of the tank could improve agitation. To confirm this fact, comparative tests of product concentration/deposition would be necessary considering agitation systems that generate different directions of fluid flow. 

### 2.5. Efficiency of the Agitation System

A test was carried out to analyse the efficiency of the agitation system. Copper oxychloride was added to the tank at a theoretical concentration of 0.4%. The sprayer was regulated at a pressure of 10 bar and the number of activated nozzles was set to 2 or 4. For each number of nozzles, five samples of 20 mL were taken at the outlet of the sprayer with the following volumes in the tank: 1000 L, 2000 L, and 3000 L. A total of 30 samples were collected. The concentration of copper oxychloride for each sample was measured by weighing after drying with precision scale at a temperature of 105 °C.

## 3. Results and Discussion

### 3.1. Effect of Independent Variables on Fluid Velocity

Considering the entire data set, the absolute velocity of the water varied between 0.69 and 37.37 cm/s. The mean velocity was 11.22 cm/s with a standard deviation of 5.95 cm/s.

The fluid velocity variable did not show a normal distribution based on the results of the Kolmogorov-Smirnov test (K-S = 0.080; *p* < 0.001). Therefore, to analyse the effects of regulation parameters of the agitation system (independent variables) on water velocity at the different measuring points inside the tank, the nonparametric Kruskal-Wallis test (SPSS Statistics v22, IBM, Armonk, NY, USA) was used. In this sense, the main independent variables of regulation of the agitation system were level of water inside the tank (1000, 2000, or 3000 L), the pressure of the nozzle circuit (8, 10, or 12 bar), and the number of nozzles working simultaneously (2 or 4). 

The fluid velocity values corresponded to the resulting absolute velocities of the three Cartesian coordinates (module of the velocity vector) measured by the ADV. Table 4 shows the results of the Kruskal-Wallis test in relation to the effect of the main independent variables on the fluid velocity. All variables had a significant effect on fluid velocity.

**Figure 5:**
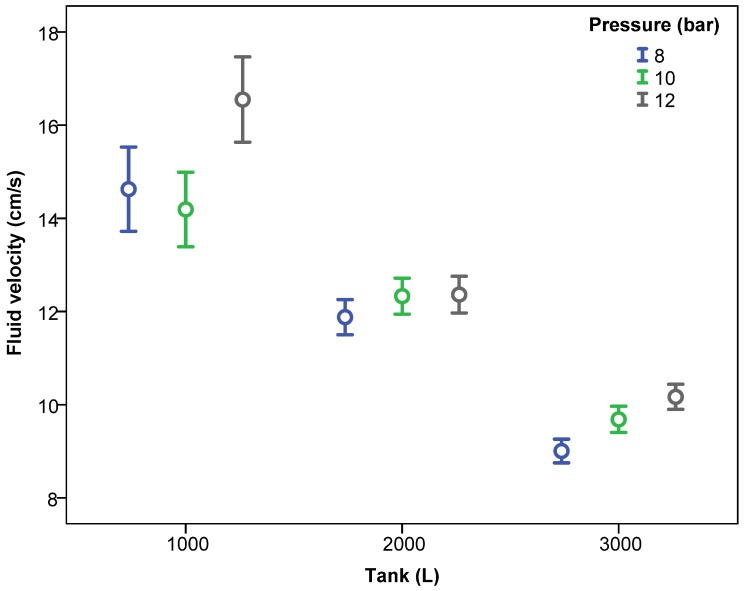
Fluid velocity (mean ± 95% confidence interval) according to the level of water in the tank and pressures of the nozzle circuits.

Considering the effect of the level of water inside the tank (1000, 2000 and 3000 L) and the pressure of the agitation circuit, Figure 5 shows the mean fluid velocity according to the level of water and the pressure. The fluid velocity decreased significantly as the water level in the tank increased. Considering mean values of all data, the fluid velocities for 1000, 2000 and 3000 L were 15.18, 12.19, and 9.61 cm/s, respectively. This fact shows that the lowest velocity values were produced when the tank was full. Results are in accordance with those obtained by [23] who used an ADV velocimeter in an aquaculture circular tank and, considering similar inlet flow rates, obtained higher velocities with the lower levels of water in the tank.

**Figure 6:**
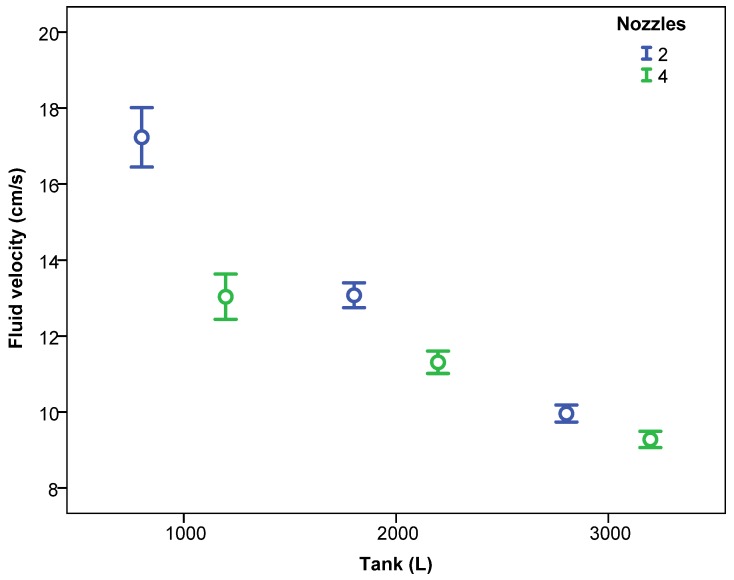
Fluid velocity (mean ± 95% confidence interval) according to the level of water in the tank and the number of active nozzles.

This fact must be considered in future design and validation phases in such way that the most demanding conditions for the agitation system occur when the tank is full. This effect was repeated for the different working pressures (Figure 5) and for the different number of nozzles of the agitation system (Figure 6). 

The pressure of the agitation circuit also had a significant influence on the fluid velocity, with higher velocities generally occurring as the pressure increased (Figure 5). These data were consistent with those obtained by [10], who measured fluid velocity using a hot-film anemometer at nine points inside a sprayer tank of 1136 L, working with four nozzles, and registered fluid velocity increments between 40% and 130% as the system pressure increased from 2.07 to 4.70 bar.

The number of active nozzles in the agitation system also significantly affected the fluid velocity (Figure 6); with 4 nozzles activated the velocities were lower than those with 2 nozzles (11.95 vs. 10.45 cm/s). This fact reflects that the location of the nozzles within the tank can affect the fluid velocity more significantly than does the number of nozzles activated. In this case, the nozzles were located on opposite sides of the tank so the effect of increasing the number of nozzles did not result in an increase in velocity. However, [10], who worked with 8 nozzles in the agitation system, registered a 14.8% velocity increase compared with that for 4 nozzles, although in this case all of the nozzles were aligned in the lower part of the tank, placed on the same work plane. This fact reinforces the importance of properly locating nozzles inside the tank, and the usefulness of velocity measurement systems, such that tested here, to validate this location.

### 3.2. Effect of Measurement Point Position

The position of the measuring point inside the tank had a significant influence on the fluid velocity. Table 5 shows the results of the Kruskal-Wallis test in relation to the effect of measuring point position (section, height, and measurement point) on the fluid velocity. All variables had a significant effect on fluid velocity.

**Figure 7:**
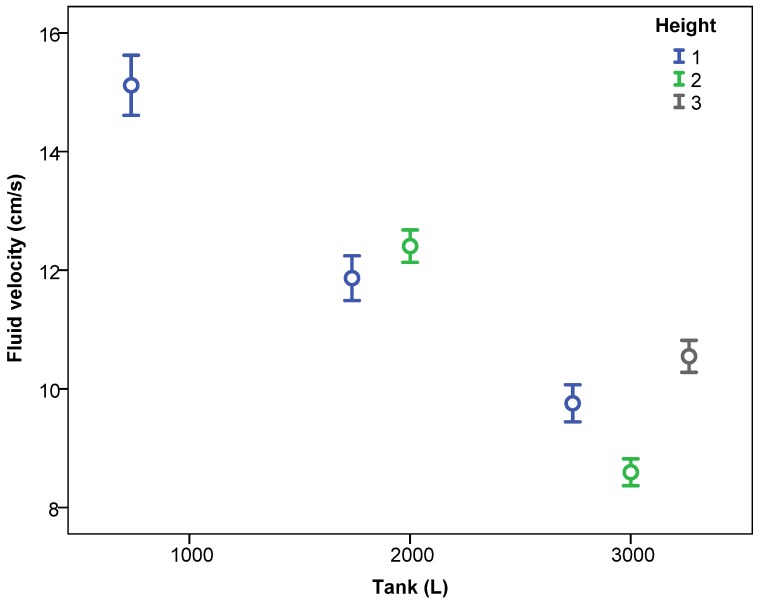
Fluid velocity (mean ± 95% confidence interval) according to the level of water in the tank and the height of the measurement point.

**Figure 8:**
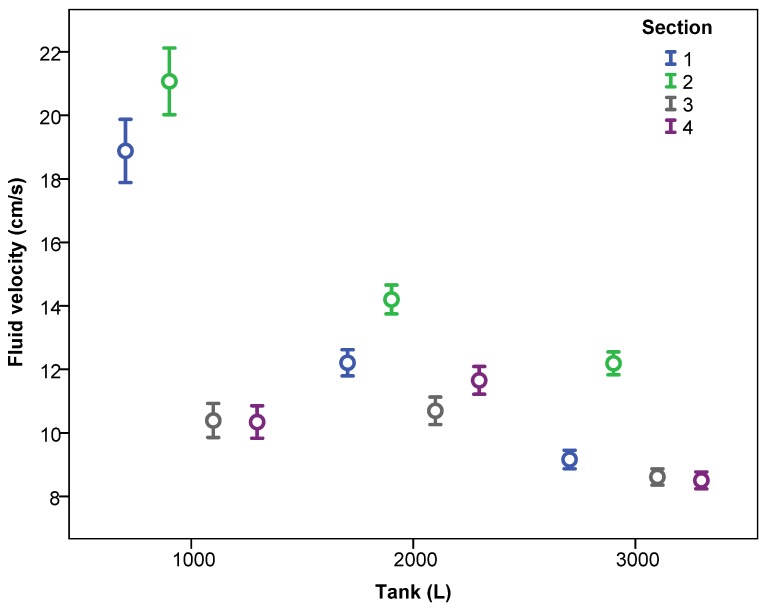
Fluid velocity (mean ± 95% confidence interval) according to the level of water in the tank and the section of the measurement point.

Considering the system as a function of the tank fill level, for all working pressures and number of nozzles, Figure 7 and Figure 8 show the fluid velocity as a function of the height and the section. There was no repetitive pattern in relation to the effect of the measurement height. Thus, for 2000 L capacity, height 2 recorded higher velocity values than did height 1, a trend that was reversed when the tank was full. In relation to the measurement section, Sections 3 and 4 had lower velocity values compared with Sections 1 and 2. In this case, the behaviour was repetitive, regardless of the tank fill level, with Section 2 having the highest velocities, followed by Section 1, then Sections 3 and 4. Thus, the influence of inner tank partitioning (Figure 2) on the recorded velocity values was clear.

**Figure 9:**
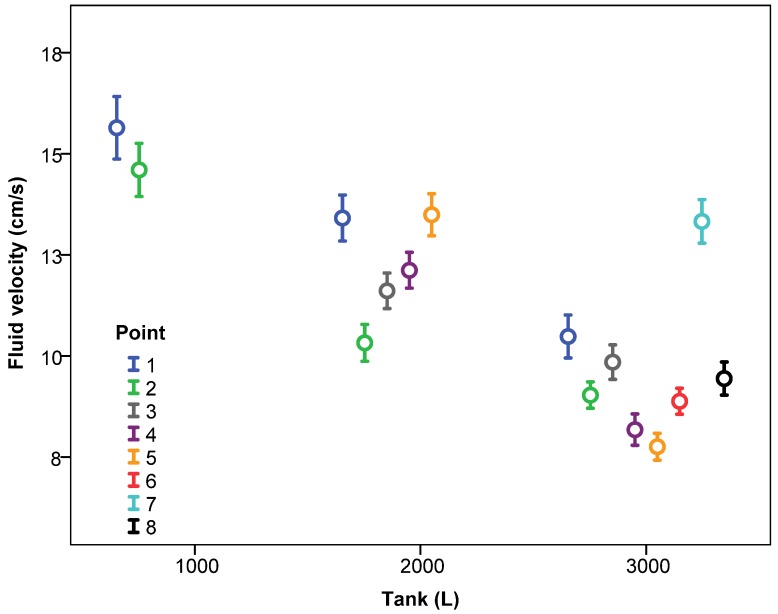
Fluid velocity (mean ± 95% confidence interval) at the measurement points of the four sections (all data included).

Considering the eight measuring points for each section (Figure 9), the behaviour was very variable; in general, for any configuration of the system, most of the points belonging to the lower zones (1 and 2 for height 1) and upper zones (7 and 8 for height 3) showed more variable behaviour; moreover, this behaviour was different for the different sections. In general, measuring points belonging to intermediate heights (3, 4, and 5) maintained more similar velocity values independent of the measuring section.

### 3.3. Efficiency of the Agitation System

The level of water inside the tank had a significant influence on the concentration of copper oxychloride. However, the effect of the number of activated nozzles was not significant. Table 6 shows the results of the univariate general linear model developed (SPSS Statistics v22) considering a significance level of 0.05.

**Figure 10:**
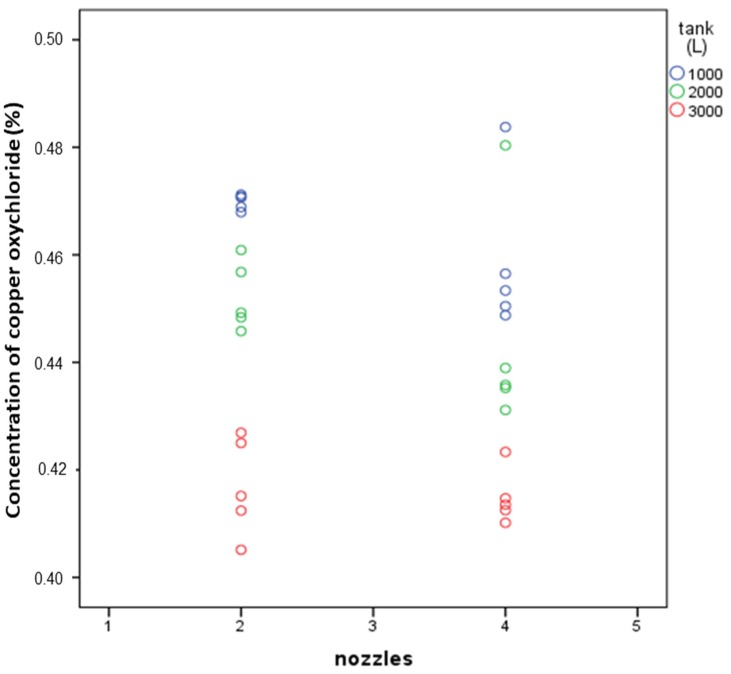
Concentration of copper oxychloride as a function of the number of nozzles and the level of water in the tank. Pressure of the agitation system: 10 bar.

The average copper oxychloride concentrations were 0.446% for 2 nozzles and 0.439% for 4 nozzles. Although these differences were not significant, there was a tendency to obtain lower concentrations of active matter with 4 nozzles for each of the three tank fill levels (1000, 2000, and 3000 L), as shown in Figure 10.

Table 7 shows the average velocities (considering all measurement points) and concentrations of copper oxychloride for tank fill levels of 1000, 2000, and 3000 L, and for two or four nozzles at a working pressure of 10 bar. The velocity values show a clear correlation with copper oxychloride concentration (linear correlation, R^2^ = 0.87; polynomial correlation, R^2^ = 0.96), with higher velocity values associated with higher concentrations. The coefficient of variation for the oxychloride concentration was 5.07%, which is a good value showing a great uniformity, for all fill level and nozzle number configurations. This indicates that the working velocity range for 10-bar regulation (9.38 to 15.96 cm/s) was sufficient to ensure a suitable mixture in the tank.

Xiongkuy et al. [24] concluded that the efficacy of agitation was improved by increases in both flow rate and working pressure; similar conclusions were obtained by other researchers [5]. In turn, it can be concluded that an increase in fluid velocity will produce an improvement in the efficacy of the agitation system.

Our tests showed higher concentrations of active matter for higher fluid velocities within the tank. This fact could indicate a greater ability of the system to mobilize active matter when fluid velocities are higher, reducing the deposition. However, differences in concentrations were low (Table 7) and further tests must be carried out to validate the results.

Independently, ADV velocimeters appears as a useful tool to set minimum velocity levels to guarantee acceptable coefficients of variation in the values of active matter concentration. This would require previous tests of active matter concentration, which, once carried out, would serve as correlation data for the practical use of ADV sensors as an indirect tool to estimate the efficiency of the agitation system.

## 4. Conclusions

ADV technology was successfully used to measure fluid velocities inside the tank of an agricultural sprayer. This technology has made it possible to evaluate the effect of sprayers’ hydraulic agitation system control parameters (i.e., water level inside the tank, hydraulic circuit pressure, and number of active nozzles) on fluid velocity. ADV is able to measure the flow speed near the bottom and boundaries of the tank because the total height of the sampling volume of the sensor is 9 mm.

All control parameters had a significant influence on the fluid velocity. Fluid velocities increased with increasing working pressure, but decreased as the tank fill level increased and as the number of active nozzles increased. In addition, the concentration of active matter in the fluid increased with increase of fluid velocity.

ADV technology makes it possible to validate the design of agitation systems in agricultural sprayers and to estimate the efficiency of these systems in order to guarantee the concentration of active matter in the fluid.

## Figures and Tables

**Table 1:** Nominal flow rate provided by a single nozzle of the agitation system at different working pressures.

Pressure (bar)	Flow Rate (L/min)
8	8.48
10	9.52
12	10.41

**Table 2:** Technical characteristics of the 3D Sontek Micro acoustic Doppler velocimeter (ADV) 16 MHz.

Parameter	Configuration
Sampling rate (Hz)	0.1 to 50
Sampling volume (cm^3^)	0.09
Distance to sampling volume (cm)	5.0
Resolution (cm/s)	0.01
Programmable velocity range (cm/s)	3, 10, 30, 100, 250
Accuracy	1% of measured velocity, ± 0.25 m/s

**Table 3:** Factorial design of experimental velocity measurements inside the tank according to the configuration variables and the location of the measurement.

Level of Water Inside the Tank (L)	Pressure (bar)	Number of Nozzles	Measurement Section (Figure 2)	Measurement Points in Each Section (Figure 2)	Height of the Measurement Point
1000	8, 10, 12	2, 4	s1, s2, s3, s4	1, 2	h1
2000	8, 10, 12	2, 4	s1, s2, s3, s4	1, 2, 3, 4, 5	h1, h2
3000	8, 10, 12	2, 4	s1, s2, s3, s4	1, 2, 3, 4, 5, 6, 7, 8	h1, h2, h3

**Table 4:** Kruskal-Wallis test. Effect of the independent variables (level of water inside the tank; pressure of the nozzle circuit; number of nozzles working simultaneously) on the fluid velocity.

Independent Variable	Settings	Dependent Variable	Chi-Square	Degrees of Freedom	Significance Level
Level of water (L)	1000, 2000, 3000	Fluid velocity	630.226	2	<0.001
Pressure (bar)	8, 10, 12		40.601	2	<0.001
Number of nozzles	2, 4		99.886	1	<0.001

**Table 5:** Kruskal-Wallis test. Effect of the variables related to the position of the measurement point (section, height, measurement point) on the fluid velocity.

Independent Variable	Settings	Dependent Variable	Chi-Square	Degrees of Freedom	Significance Level
Measurement section	1, 2, 3, 4	Fluid velocity	575.110	3	<0.001
Height of the measurement point (mm)	1 (370) 2 (650) 3 (930)	Fluid velocity	78.422	2	<0.001
Point of measurement	1, 2, 3, 4, 5, 6, 7, 8	Fluid velocity	286.155	7	<0.001

**Table 6:** Univariate general linear model of the concentration of copper oxychloride according to the level of water into the tank (1000, 2000, 3000 L) and the number of nozzles (2, 4).

Origin	Sum of Squares	Degrees of Freedom	Root Mean Square	F	Significance
Revised model	0.013a	5	0.003	19.663	<0.001
Interception	5.882	1	5.882	45,798.799	<0.001
Tank level	0.012	2	0.006	47.252	<0.001
Nozzles	0.000	1	0.000	2.954	0.099
Tank* nozzles	0.000	2	5.487 × 10^−5^	0.427	0.657
Error	0.003	24	0.000		
Total	5.898	30			
Total corrected	0.016	29			

a R^2^ = 0.804 (Adjusted R^2^ = 0.763).

**Table 7:** Fluid velocity inside the tank and concentration of copper oxychloride at the outlet of the sprayer as a function water level inside the tank and the number of activated nozzles.

Pressure (bar)	Level of Water Inside the Tank (L)	Number of Nozzles	Fluid Velocity (cm/s)	Copper Oxychloride Concentration (%)	Variation of Copper Oxychloride Concentration (%)
10	1000	2	15.9693	0.4699	17.45
		4	12.4986	0.4586	14.65
	2000	2	13.1345	0.4522	13.05
		4	11.5460	0.4443	11.07
	3000	2	9.9866	0.4169	4.22
		4	9.3831	0.4148	3.70

## References

[1] Ali B., Ali B. (2017). Performance of a hydraulic jet agitation system with different jet nozzle sizes in the sprayer tank. Agronomy.

[2] ISO (1997). Equipment for Crop Protection—Spraying Equipment—Part 2: Test Methods for Hydraulic Sprayers.

[3] ISO (2015). Agricultural and Forestry Machinery—Inspection of Sprayer and Liquid Fertilizer in Use; Part 1. General; Part 2. Boom Sprayers; Part 3. Sprayers for Bush and Trees.

[4] Balsari P., Tamagnone M., Allochis D., Marucco P., Bozzer C. Sprayer tank agitation check: A proposal for a simple instrumental evaluation. Proceedings of the Fourth European Workshop on Standardised Procedure for the Inspection of Sprayers—SPISE 4.

[5] Tamagnone M., Balsari P., Bozzer C., Marucco P. (2012). Assessment of parameters needed to design agitation systems for sprayer tanks. Asp. Appl. Biol..

[6] Ozkan H.E., Ackerman K.K. (1999). Instrumentation for measuring mixture variability in sprayer tanks. Appl. Eng. Agric..

[7] Ucar T., Ozkan H.E., Fox R.D., Brazee R.D., Derksen R.C. (2000). Experimental study of jet agitation effects on agrochemical mixing in sprayer tanks. J. Agric. Eng. Res..

[8] Vondricka J., Schulze P. (2009). Measurement of mixture homogeneity in direct injection systems. Trans. ASABE.

[9] Armenante P.M., Luo C., Chou C., Fort I., Medek J. (1997). Velocity profiles in a closed unbaffled vessel: Comparison between experimental LDV data and numerical CFD predictions. Chem. Eng. Sci..

[10] Ucar T., Fox R.D., Ozkan H.E., Brazee R.D. (2001). Simulation of jet agitation in sprayer tanks: Comparison of predicted and measured water velocities. Trans. ASABE.

[11] Chen N., Liao B., Pan J., Li Q., Gao C. (2006). Improvement of the flow rate distribution in quench tank by measurement and computer simulation. Mater. Lett..

[12] Micheli G.B., Padilha A., Scalon V.L. (2015). Numerical and experimental analysis of pesticide spray mixing in spray tanks. Eng. Agric. Jaboticabal..

[13] García C.M., Cantero M.I., Niño Y., García M.H. (2005). Turbulence measurements with acoustic Doppler velocimeters. J. Hydraul. Eng..

[14] Poindexter C.M., Rusello P.J., Variano E.J. (2011). Acoustic Doppler velocimeter-induced acoustic streaming and its implication for measurement. Exp. Fluids.

[15] MacVicar B.J., Beaulieu E., Champagne V., Roy A.G. (2007). Measuring water velocity in highly turbulent flows: Field tests of an electromagnetic current meter (ECM) and an acoustic Doppler velocimeter (ADV). Earth Surf. Process. Landforms.

[16] Hosseini S.A., Shamsai A., Ataie-Ashtiani B. (2006). Synchronous measurements of the velocity and concentration in low density turbidity currents using an acoustic Doppler velocimeter. Flow Meas. Instrum..

[17] Sharma A., Maddirala A.K., Kumar B. (2018). Modified singular spectrum analysis for despiking acoustic Doppler velocimeter (ADV) data. Measurement.

[18] Liao Q., Wang B., Wang P.F. (2015). In situ measurement of sediment resuspension caused by propeller wash with an underwater particle image velocimetry and an acoustic Doppler velocimeter. Flow Meas. Instrum..

[19] Sontek/YSI (2001). FlowTracker Handheld ADV.

[20] Durgesh V., Thomson J., Richmond M.C., Polagye B.L. (2014). Noise correction of turbulent spectra obtained from acoustic Doppler velocimeters. Flow Meas. Instrum..

[21] Voulgaris G., Trowbridge J. (1998). Evaluation of the acoustic Doppler velocimeter (ADV) for turbulence measurements. J. Atmos. Ocean. Tech..

[22] Wahl T.L. Analyzing ADV data using WinADV. Proceedings of the Joint Conference on Water Resources Engineering and Water Resources Planning & Management.

[23] Oca J., Masalo I. (2013). Flow pattern in aquaculture circular tanks: Influence of flow rate, water depth, and water inlet & outlet features. Aquacult. Eng..

[24] Xiongkuy H., Kleisinger S., Luoluo W., Bingli L. (1999). Influences of dynamic factors and filling level of spray in the tank on the efficacy of hydraulic agitation of the sprayer. T. Chin Soc. Agric. Eng..

